# Cardiopulmonary Exercise Testing as a Predictor of Postoperative Outcome in Patients Undergoing Oesophageal Cancer Surgery Following Neoadjuvant Chemotherapy

**DOI:** 10.5152/TJAR.2022.21158

**Published:** 2022-10-01

**Authors:** Aditi Suri, Seema Mishra, Sushma Bhatnagar, Rakesh Garg, Sachidanant Jee Bharti, Vinod Kumar, Nishkarsh Gupta, Sunil Kumar, Atul Sharma, Suryanarayana Deo

**Affiliations:** 1 Department of Onco-Anaesthesia and Palliative Medicine, Dr. BRA-IRCH, All India Institute of Medical Sciences, New Delhi, India; 2 Department of Surgical Oncology, Dr. BRA-IRCH, All India Institute of Medical Sciences, New Delhi, India; 3 Department of Medical Oncology, Dr. BRA-IRCH, All India Institute of Medical Sciences, New Delhi, India

**Keywords:** Anaerobic threshold, cardiopulmonary exercise testing, Clavien–Dindo classification, neoadjuvant chemotherapy, oesophagectomy, oesophagus cancer

## Abstract

**Objective:**

Neoadjuvant chemotherapy improves resectability rates of oesophageal cancer, but the process may also take a toll on the patients’ exercise capacity and may adversely affect the postoperative outcomes. It can be assessed objectively using cardiopulmonary exercise testing.

**Methods:**

Patients with oesophagus cancer performed a baseline test and a second test after neoadjuvant chemotherapy during the week preceding oesophagectomy. They were followed up for postoperative complications, length of hospital stay, and 30-day mortality.

**Results:**

Thirty-three patients completed the study. The mean pre-chemotherapy peak oxygen uptake (VO_2_ peak) was 1128.39 ± 202.79 mL min^−1^ (19.46 ± 3.06 mL kg^−1^ min^−1^) which declined to 1010.33 ± 195.56 mL min^−1^ (17.24 ± 2.55 mL kg^−1^ min^−1^) in the post-chemotherapy period (*P *< .001). Pre-chemotherapy anaerobic threshold was 906.85 ± 176.81 mL min^−1^ (15.54 ± 2.24 mL kg^−1^ min^−1^) which declined to 764.76 ± 158.79 mL min^−1^ (13.01 ± 2.22 mL kg^−1^ min^−1^) (*P* < .001) in the post-chemotherapy period. Six patients developed complications of modified Clavien–Dindo grade 3 and above. Two (6.1%) patients succumbed to complications within 30 days. The mean anaerobic threshold in patients who suffered complications modified Clavien–Dindo grade ≥3 was 693.33 ± 140.99 mL min^−1^ (11.2 ± 1.17 mL kg^−1^ min^−1^) while patients with mild to moderate complications had a mean anaerobic threshold 13.41 ± 2.21 mL kg^−1^ min^−1^ (*P* < .006). An optimal cut off value for anaerobic threshold was 12.5 mL kg^−1^ min^−1^.

**Conclusion:**

Cardiopulmonary exercise testing accurately predicts outcomes in cancer oesophagus patients who undergo neoadjuvant chemotherapy followed by surgery.

**Main Points**

- Neoadjuvant chemotherapy causes a decline in functional capacity.
- Cardiopulmonary exercise testing objectively measures functional reserve.
- Anaerobic threshold significantly declines after chemotherapy.
- Poor anaerobic threshold correlates with poor postoperative outcomes.

## Introduction

Oesophageal cancer is one of the most aggressive cancers worldwide with a poor survival rate. It is the eighth most common cause of cancer-related deaths.^1,2^ Oesophagectomy offers a curative potential for oesophageal cancer, but being a challenging procedure, it has 30-day mortality of around 0.5%-12%.^3,4^ A variety of factors potentially influence treatment outcomes, like the effects of chemotherapy, nutritional status, and general overall fitness. Neoadjuvant chemotherapy followed by surgery improves resection rates.^5-7^ But Neoadjuvant Chemotherapy (NACT) too can take a toll on the patient’s cardiopulmonary reserve, causing a substantial decline in exercise capacity.^8^ This is a result of proteolysis, skeletal muscle wasting, and oxidative damage.^6,9,10^ This decrement in functional capacity may or may not return to baseline values by the time of surgery.

The utility of cardiopulmonary exercise testing (CPET) has been studied extensively for the prediction of postoperative morbidity. Being an objective and dynamic test, it is the “gold standard” test to assess integrated functions of the cardiovascular, respiratory, metabolic, and hematological systems.^11-13^ Perioperative Exercise Testing and Training Society (POETTS) guidelines recommend the use of CPET variables for risk stratification in surgeries associated with morbidity as well as for assessing the effects of neoadjuvant therapies.^14^ Most commonly studied CPET variables are anaerobic threshold (AT) and peak oxygen uptake (VO_2_ peak). Lower values of AT and VO_2_ peak have been found to be associated with adverse postoperative outcomes in rectal surgeries.^15^ Previous studies that explored the role of CPET in predicting outcome after oesophageal cancer surgery found a significant correlation between CPET variables and postoperative outcome.^16,17^ Only a few authors have evaluated the effect of neoadjuvant chemotherapy on cardiopulmonary fitness with variable results.^16,18,19^

The present study, therefore, aimed to evaluate whether CPET variables can predict postoperative outcomes in oesophageal cancer surgery at a tertiary care center in India. A null hypothesis of “CPET is not a predictor of postoperative outcome in patients undergoing NACT followed by oesophagectomy” was established. An alternate hypothesis stating that “CPET is a predictor of postoperative outcome in patients undergoing NACT followed by oesophagectomy” was formulated.

The primary objective was to study the correlation between CPET variables and postoperative morbidity in patients with oesophagus cancer undergoing neoadjuvant chemotherapy followed by surgery. We also aimed to compare pre-chemotherapy and post-chemotherapy CPET variables and their association with postoperative outcome, as well as the association of CPET variables with the length of hospital stay (LOHS) and 30-day mortality.

## Methods

This prospective observational study was performed after receiving approval from the Institutional Ethics Committee (IECPG-297/28.6.2018). Written informed consent was obtained from every patient before their enrolment into the study. Patients of age 18-75 years, belonging to American Society of Anesthesiologists (ASA) class I and II, suffering from oesophagus cancer (squamous cell carcinoma [SCC] or adenocarcinoma [AC]) of the middle or lower third of the oesophagus, and undergoing neoadjuvant chemotherapy followed by transthoracic Mckeown’s or Ivor Lewis oesophagectomy were included in our study after obtaining written informed consent. All procedures were conducted in accordance with the Helsinki Declaration-2013.

All patients presenting with oesophagus cancer to our thoracic oncology clinic were thoroughly assessed by surgical oncologists. Patients were asked to perform a baseline CPET before the onset of chemotherapy, followed by chemotherapy as per standard institutional protocol. The chemotherapeutic agents administered were either 5-fluorouracil and cisplatin or paclitaxel and carboplatin, or FLOT regime (5-fluorouracil, leucovorin, oxaliplatin, docetaxel) as per the medical oncologists’ institutional protocol.^20^ The same patients were followed up and the second session of CPET was performed within the week preceding surgery. Patients were taken up for surgery around 6-8 weeks after chemotherapy. They were then assessed for postoperative outcomes using Post Operative Morbidity Survey (POMS) on days 3, 5, and 7, and the modified Clavien–Dindo (CD) classification (Supplementary Table 1) scores from 0 to 5 were used to assess severity.^21,22^ Any in-hospital mortality was also recorded. All patients were followed until the 30-day post-procedure to note the 30-day mortality. Postoperative intensive care unit (ICU) admission, length of ICU stay, and LOHS were all duly recorded.

### CPET Protocol

Cardiopulmonary exercise testing was performed as per American Thoracic Society/American College of Chest Physicians (ATS/ACCP) recommendations.^11^ All patients were asked to abstain from smoking for at least 8 hours prior to the procedure, take a light breakfast 2 hours before the test, refrain from exercise on the day of the test, and take usual medications as prescribed. Flow and gas calibrations were performed before each test. A brief history of the patient’s co-morbidities and medications was taken along with a brief physical examination, and the diagnostic test results were noted. A 12-lead electrocardiogram, pulse oximetry, and non-invasive blood pressure were monitored throughout the procedure. Exercise testing was conducted on an electromagnetically braked cycle ergometer (Ergoline, Lindenstrasse, Germany) and breath by breath gas exchange measurements were performed using a metabolic cart (Quark CPET, COSMED, Rome, Italy). The maximal incremental cycle ergometry protocol was conducted as follows: (1) Unloaded “freewheel” cycling @ 55-65 rpm for 3 minutes. (2) Cycling against incremental workload until the patient reported exhaustion. The rate of increase of workload was 5-25 Watt/min based on patient criteria, derived from a formula recommended by the ATS/ACCP. (3) Ten minutes of recovery consisting of 3 minutes of unloaded cycling @ 60 rpm.

Cardiopulmonary exercise testing measurements including VO_2_ peak and ventilatory equivalents for carbon dioxide (V_E_/VCO_2_) were all measured at peak exercise and at anaerobic threshold (AT). The AT was non-invasively measured using a combination of the V-slope and the ventilatory equivalents methods. All tests were performed by 1 anaesthesiologist and were reported independently by 2 experienced anaesthesiologists. Any discrepancy was resolved by a third anaesthesiologist. All CPET exercises were conducted in the presence of an anaesthesiologist while continually monitoring vitals and ECG for signs of ischemic changes. Assessment of maximal patient effort was done in compliance with the ATS/ACCP recommendation. The test was prematurely terminated in the event of chest pain suggestive of ischemia, arrhythmia, fall in systolic blood pressure (SBP) >20 mm Hg, hypertension >250 mm Hg SBP and >120 mm Hg diastolic blood pressure (DBP), oxygen desaturation: ΔSpO_2 _>5%, sudden pallor, loss of coordination, mental confusion, dizziness, or faintness.^11^

## Sample Size Calculation

With reference to a previous study published by Navidi et al^16^ the observed mean AT at baseline and at 4 weeks was 15.3 ± 3.4 mL^−1^ kg^−1^ min^−1^ and 12.6 ± 2.7 mL^−1^ kg^−1^ min^−1^, respectively. Taking these values as a reference, the minimum required sample size with 90% power of study and 5% level of significance is 26 patients. Considering patients lost to follow-up as 20%, the total sample size was taken as 33.

## Formula for comparing mean of pre-chemotherapy and post-chemotherapy AT was:



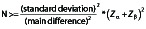



Where Z_α _is value of Z at 2-sided alpha error of 5% and Z_β_ is value of Z at power of 90% and mean difference is difference in mean values of pre and post.



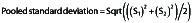

- S_1_ is standard deviation of pre-chemotherapy AT.
- S_2_ is standard deviation of post-chemotherapy AT.

## Anaerobic threshold calculated with the below formula:



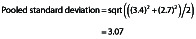





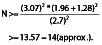



Nevertheless, all consecutive patients presenting with oesophageal cancer to our hospital between a period from September 2018 to January 2019 and those meeting inclusion criteria were included in the study.

### Statistical Analysis

Qualitative variables were expressed as absolute and relative frequencies and continuous variables were depicted as mean (standard deviation). The correlation was analyzed using Spearman’s rank correlation. To compare qualitative variables, the chi-square test or Fisher’s exact test and Wilcoxon test was used for quantitative variables according to the distribution of data. Receiver operating curves (ROC) were used to determine a threshold for continuous data and also to determine sensitivity and specificity. To determine the factors associated with a binary outcome, logistic regression analysis was used. A *P*-value of less than .05 was considered statistically significant. Statistical analysis was performed using Statistical Package for the Social Sciences software 22 (IBM Corp.; Armonk, NY, USA).

## Results

**Figure 1.**
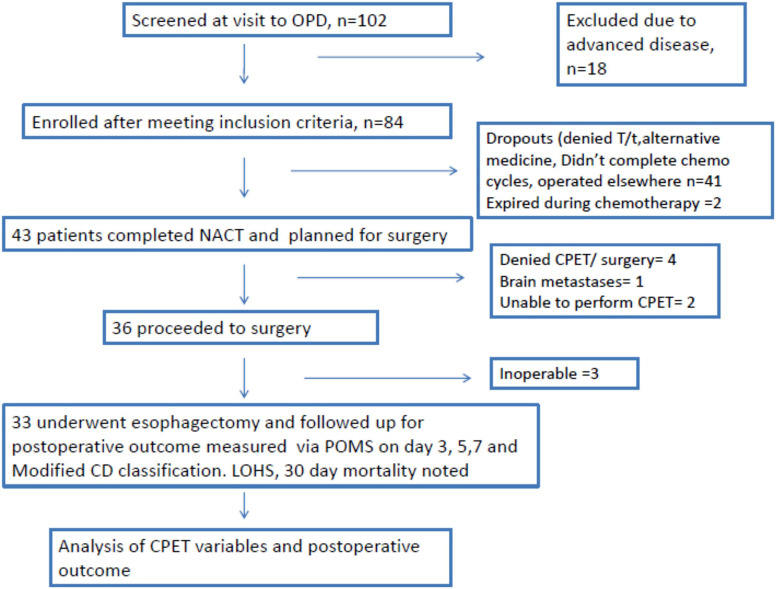
Flow diagram of the study. CPET, cardiopulmonary exercise testing; NACT, neoadjuvant chemotherapy; POMS, postoperative morbidity survey; CD, Clavien–Dindo; LOHS, Length of hospital stay.

A total of 102 patients were screened, of which, 84 were enrolled in the study. Forty-three patients out of these were able to complete neoadjuvant chemotherapy and were deemed fit for surgery. Only 33 patients out of these got operated and could complete the study (Figure 1).

The mean age was 56 ± 9.81 years. There were 13 females and 20 males. Ten patients belonged to ASA class I, while 19 (56.6%) and 4 (12.1%) belonged to ASA classes 2 and 3, respectively. The mean pre-chemo body mass index was 22 ± 4.26 Kg m^-2^. Twenty-five (75%) patients were diagnosed to have SCC while AC was the histological diagnosis in 8 (24.2%) patients. Tumor location was the middle third of the oesophagus in 14 (42.4%) cases, while it was the lower third in 19 (57.6%) cases. Ivor Lewis oesophagectomy was performed in 51.5% of patients and 48.5% of patients underwent Mckweon’s oesophagectomy. The mean pre-chemotherapy VO_2 _peak and AT as well as their subsequent decline after chemotherapy are summarized in (Table 1). The pre-chemotherapy AT was 906.85 ± 176.81 mL min^−1^ (15.54 ± 2.24 mL kg^−1^ min^−1^) which later declined significantly to 764.76 ± 158.79 mL min^−1^ (13.01 ± 2.22 mL kg^−1^ min^−1^) (*P* < .001) in the post-chemotherapy period. The mean decline of AT in the pre- and post-chemotherapy values was observed to be 2.52 mL kg^−1^ min^−1^. Similarly, the mean fall in VO_2_ peak was 2.2 mL kg^−1^ min^−1^, which too was statistically significant (*P* < .001).

As observed using POMS,^21^ the most common postoperative complications seen were pulmonary in 20 (60.6 %), 17 (21.2%), and 4 (12.1%) patients on days 3, 5, and 7, respectively, ranging from pleural effusion, pneumothorax, and bronchospasm. Two patients developed an anastomotic leak, out of which, 1 was a major leak, thence re-explored while the other was a minor leak, and was managed conservatively. Cardiovascular complications like atrial arrhythmias, ischemic changes, or prolonged vasopressor support were noted in 7 (21.2%) patients at postoperative day (POD) 3. Neurological complications like recurrent laryngeal nerve palsy were observed in 1 patient that was managed conservatively. One patient developed acute kidney injury needing an ICU stay of 4 days. Four patients required readmission to ICU. One patient with preexisting basilic vein thrombosis developed sudden onset of severe respiratory distress and hemodynamic instability on POD3. He was intubated and shifted to ICU but passed away eventually on POD4. Out of the other 3 patients who were readmitted to ICU, 2 were intubated, 1 of which was later extubated and discharged and 1 was tracheostomized and later passed away due to sepsis, and 1 patient needed supplemental oxygen only and was observed in ICU for a day and discharged.

**Figure 2.**
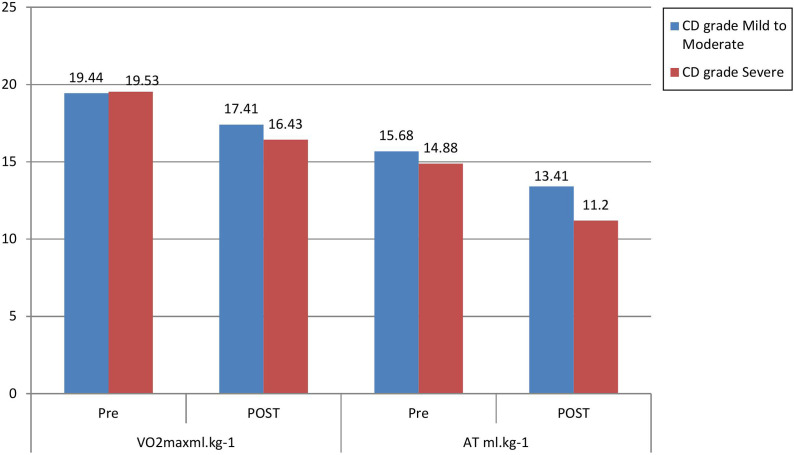
Figure displays comparison of exercise testing variables with severity of complications. Variables are depicted as means. AT, anaerobic threshold; CD, Clavien–Dindo.

Six patients developed complications of modified CD grade 3 and above (Table 2). Two (6.1%) patients succumbed to complications within 30 days. The mean LOHS was 14.7 ± 5.94 (7-32) days. The mean AT in patients who suffered complications of modified CD grade ≥3 was 693.33 ± 140.99 mL min^−1^ (11.2 ± 1.17 mL kg^−1^ min^−1^) while patients who suffered mild to moderate complications had a mean AT of 13.41 ± 2.21 mL kg^−1^ min^−1^ (*P* < .006). A significant correlation with AT and severe postoperative complications was observed (Tables 3 and 4). A lower value of VO_2_ peak was not found to be associated with an increased risk of cardiopulmonary complications (16.43 ± 2.43 mL kg^−1^ min^−1^, *P *= .403). The ROC curves for AT and postoperative complications are shown in Figure 2. The cutoff AT value as derived from the ROC was 12.5 mL kg^−1^ min^−1^ with a sensitivity and specificity of 74.0% and 66.67%, respectively. The sensitivity and specificity observed for an AT of 11 mL kg^−1^ min^−1^ were 66.7% and 85.2%, respectively. It had a positive predictive value of 50% and a negative predictive value of 92%.

**Figure 3.**
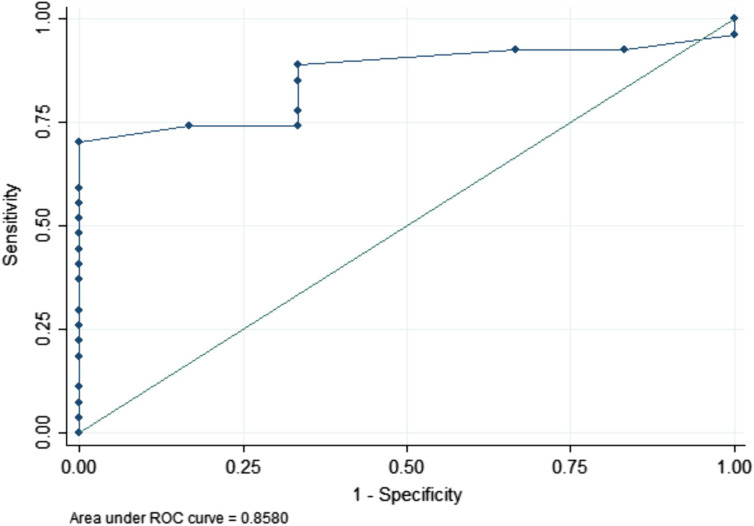
ROC curve for post-chemotherapy AT and outcome. ROC, receiver operating characteristic curve; AT, anaerobic threshold.

Spearman’s correlation coefficient was used to assess the correlation between AT and LOHS (Figure 3). No significant correlation was found between VO_2_ peak and AT with 30-day mortality. Univariate analysis of preoperative demographic and exercise test variables is shown in Table 5. Ventilatory equivalents for carbon dioxide (VE/VCO_2_) at AT did not have a statistically significant relationship with the postoperative outcome (*P*  = .304). No significant correlation was found between tumor location, histological type, chemotherapy regime, surgical approaches, 30-day mortality, and the outcome.

## Discussion

Cardiopulmonary exercise testing is a dynamic tool that enables the holistic evaluation of cardiopulmonary reserve. Variations in body composition, muscle conditioning, anemia, and nutritional status can lead to variable CPET performances in subjects from different demographic groups. The benefits of CPET as a perioperative diagnostic tool were first assessed by Older et al^23^ in 1993, who affirmed its utility in elderly patients undergoing major abdominal surgery for the presence of cardiac failure. They concluded that an AT of <11 mL kg^−1^ min^−1^ was associated with a higher risk of complications. Several other studies have ever since evaluated the predictive capacity of CPET variables for postoperative complications.^24-26^ But the data reported in previous literature may or may not apply to all patient populations.

Neoadjuvant chemotherapy in oesophageal cancer improves overall survival by downstaging the tumor, increasing resection rates, and treating micrometastases that are not detected in imaging studies.^7^ But the changes in fat-free mass, skeletal muscle, and body cell mass after NACT result in sarcopenia, weight loss, and a decline in functional capacity.^8,27^ These changes can be assessed objectively using CPET, thus providing valuable information on perioperative cardiopulmonary reserve.^24,28^

The mean pre-chemotherapy and post-chemotherapy VO_2_ peak observed in our study were 1128.39 ± 202.79 mL min^−1^ (19.46 ± 3.06 mL kg^−1^ min^−1^) and 1010.33 ± 195.56 mL min^−1^ (17.24 ± 2.55 mL kg^−1^ min^−1^), respectively, (*P* < .001). Similarly, the pre-chemotherapy AT declined from 906.85 ± 176.81 mL min^−1^ (15.54 ± 2.24 mL kg^−1^ min^−1^) to 764.76 ± 158.79 mL min^−1^ (13.01 ± 2.22 mL kg^−1^ min^−1^) (*P* < .001) in the post-chemotherapy period. These observations are similar to previous studies. Navidi et al^16^ too observed a significant decline in the mean AT, between baseline (15.3 mL kg^−1^ min^−1^), second (11.9 mL kg^−1^ min^−1^), third (12.1 mL kg^−1^ min^−1^), and fourth test (12.6 mL kg^−1^ min^−1^) (*P <* ⋅010). A similar pattern was observed for the VO_2_ peak (*P* < .010).

Jack et al^29^ found the VO_2_ peak and AT to have declined after NACT from 14.5 ± 3.8 mL kg^−1^ min^−1^ to 12.3 ± 3.0 mL kg^−1^ min^−1^ (*P* < .001) for AT and 20.8 ± 6.0 mL kg^−1^ min^−1^ to 18.3 ± 5.1 mL kg^−1^ min^−1^ for VO_2_ peak (*P* < .001). They concluded that a lower baseline exercise capacity was associated with higher 1-year mortality in patients completing NACT and surgery. Sinclair et al^19^ also demonstrated a decline in baseline and post-chemotherapy AT in 30 patients with oesophageal adenocarcinoma. The baseline AT decreased from 13.9 ± 3.1 mL kg^−1^ min^−1^ to 11.5 ± 2.0 mL kg^−1^ min^−1^ in post-chemotherapy period. The mean decline was 2.4 mL kg^−1^ min^−1^ (*P* < .001).

In contrast to the above, Drummond et al^18^ found the effect of NACT on AT to be insignificant (*P*  = .756). But this study was limited by an overall lower AT in the population compared to previously published cohorts and the possibility of selection bias as highlighted by the authors.

The time interval between NACT and surgery is usually kept around 6-8 weeks to facilitate recuperation from the physiological effects of chemotherapy and to achieve tumor size reduction. Navidi et al^16^ found that the decline in CPET variables after NACT was sustained till the time of surgery. Similar results were obtained in our study.

Previous literature has shown a VO_2_ peak of below 17 mL kg^−1^ min^−1^ and AT values below 10.5 mL kg^−1^ min^−1^ strongly correlates with postoperative morbidity.^17^ Our study showed a lower mean AT of 11.2 ± 1.17 mL kg^−1^ min^−1^ to be associated with severe postoperative complications (*P*  = .025). But a lower value of VO_2_ peak did not predict the risk of severe postoperative complications in our study (16.43 ± 2.43, *P  *= .403). Moyes et al^30^ too reported that a lower VO_2_ peak did not correlate with cardiopulmonary complications (14.6 mL kg^−1^ min^−1^ vs 16.6 mL kg^−1^ min^−1^, *P*  = .07).

**Figure 4.**
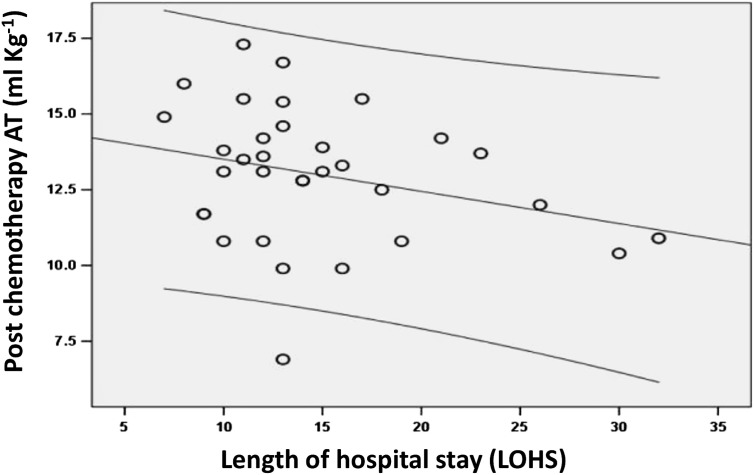
Spearman’s correlation between post-chemotherapy AT and LOHS. AT, anaerobic threshold; LOHS, length of hospital stay.

In our study, the ROC curves for AT and postoperative complications showed an area under the curve (AUC) of 0.858 (95% CI: 0.72-0.98) (Figure 4). The cutoff AT value as derived from the ROC was 12.5 mL kg^−1^ min^−1^ with a sensitivity and specificity of 74.0% and 66.67% respectively. Patel et al^17^ too obtained an optimal cutoff value of 10.5 mL kg^−1^ min^−1^ (AUC: 0.62, 95% CI: 0.51-0.74, *P*  = .048) with a sensitivity of 60% and specificity of 44%.

Our study, therefore, highlights that CPET is valuable in predicting postoperative outcomes in patients undergoing oesophagectomy. It brings to light the impact of NACT on patients’ overall fitness and postoperative outcome. The study opens a gateway for research on prehabilitation that can be instrumental in positively impacting postoperative outcomes.^14^

The strengths of our study include optimal patient care by an experienced multidisciplinary team and the application of a modified CD classification dedicated solely to the classification of post-oesophagectomy complications. This is also the first such study on the Indian patient population suffering from cancer of the oesophagus. There are several limitations to this study. Firstly, this was a non-blinded study. The perioperative care team had access to the CPET results, although the perioperative care was not affected by the results. Another shortcoming is the sample size. A large proportion of cancer oesophagus patients attending clinics at our tertiary care hospital are seen to present at an advanced stage at diagnosis itself. Two patients with bilateral knee osteoarthritis, who had been excluded from the study due to inability to perform CPET, later underwent esophagectomy uneventfully. This is a shortcoming of CPET via cycle ergometer, wherein the cardiopulmonary reserve could not be assessed objectively.

Neoadjuvant chemotherapy, therefore, holds the potential to positively or negatively impact the postoperative outcome. Anaerobic threshold, as measured by CPET, deteriorates significantly after NACT and is predictive of postoperative complications. This makes CPET a vital tool to predict postoperative outcomes after esophageal cancer surgery.

## Figures and Tables

**Supplementary Table 1.** Postoperative Morbidity Survey (POMS). Modified Clavien–Dindo (CD) Classification for Oesophagectomy

A.**Postoperative Morbidity Survey (POMS)21**			
Pulmonary	Has the patient developed a new requirement for oxygen or respiratory support		Patient observation Treatment chart
Infectious	Currently on antibiotics and/or has had a temperature of 38°C in the last 24 hours		Treatment chart Observation chart
Renal	Presence of oliguria less than 500ml/24 hrs increased serum creatinine (30% from preoperative level); urinary catheter in situ		Fluid balance chart Biochemistry result Patient observation
Gastrointestinal	Unable to tolerate an enteral diet for any reason including nausea, vomiting, and abdominal distension (use of antiemetic)		Patient questioning Fluid balance chart Treatment chart
Cardiovascular	Diagnostic tests or therapy within the last 24 hours for any of the following: new myocardial infarction or ischemia, hypotension (requiring fluid therapy 200 mL/h or pharmacological therapy), atrial or ventricular arrhythmias, cardiogenic pulmonary edema, thrombotic event (requiring anticoagulation)		Treatment chart Note review
Neurological	New focal neurological deficit, confusion, delirium, or coma		Note review Patient questioning
Hematological	Requirement for any of the following within the last 24 hours: packed erythrocytes, platelets, fresh-frozen plasma, or cryoprecipitate		Treatment chart Fluid balance chart
Wound	Wound dehiscence requiring surgical exploration or drainage of pus from the operation wound with or without isolation of organisms		Note review Pathology result
Pain	New postoperative pain significant enough to require parenteral opioids or regional analgesia		Treatment chart
A.**Modified Clavien**–**Dindo (CD) Classification for Oesophagectomy22**			
**CD grade**		**Complications**	
0		No complications	
1		Deviation from normal hospital course	
2		Requiring pharmacological treatment	
3a		Interventions not under GA	
3b		Interventions under GA	
4a		Single organ dysfunction, ICU admission	
4b		Multiple organ dysfunction, ICU admission	
5		Mortality	

GA, General anaesthesia; ICU, intensive care unit.

**Table 1.** Comparison of Pre-chemotherapy and Post-chemotherapy VO_2_ Peak and AT

	**Pre-chemotherapy**	**Post-chemotherapy**	*P*
VO_2_ peak	1128.3 ± 202.7	1010.3 ± 195.5	<.001
VO2peak (mL min−1)	19.4 ± 3.0	17.2 ± 2.5	<.001
AT (mL min−1)	906.8 ± 176.8	764.7 ± 158.7	<.001
AT (mL kg min−1)	15.5 ± 2.2	13.0 ± 2.2	<.001
Weight (kg)	58.85 ± 11.4	59.18 ± 9.44	.630
BMI (kg m−2)	22.05 ± 4.5	22.28 ± 4.14	.345

Values are mean ± SD, median (25th-75th percentile). Change in CPET variables from pre-chemotherapy to post-chemotherapy period.

VO_2_ peak, peak oxygen uptake; AT, anaerobic threshold; BMI, body mass index.

**Table 2.** Modified Clavien–Dindo (CD) Classification

**Modified CD grade**	**Frequency (%)**
0	9 (27.3)
1	15 (45.5)
2	3 (9.1)
3A	2 (9.1)
4A	2 (6.1)
5	2 (6.1)
Total	33 (100)

Values are given as percentages. Table depicts number and percentages of patients who experienced complications in accordance with modified Clavien–Dindo (CD) classification.

CD grades: 0 = no complications; 1 = deviation from normal hospital course; 2 = requiring pharmacological treatment; 3a = interventions not under GA; 3b = interventions under GA; 4a = single-organ dysfunction, ICU admission; 4b = multiple organ dysfunction, ICU admission; 5 = mortality.

**Table 3.** Relationship of Post-chemotherapy AT and Postoperative Complications

**Post AT (mL kg−1)**	**CD grade**				*P*
	**Mild to Moderate**		**Severe**		
**Frequency**	**%**	**Frequency**	**%**
≤11	4	14.8	4	66.7	.020
>11	23	85.2	2	33.3	
Total	27	100	6	100

Values are given as percentages. Table depicts percentage of patients with mild, moderate, and severe complications as a comparison to AT.

AT, anaerobic threshold; CD, Clavien –Dindo.

**Table 4.** CPET Variables and Postoperative Complications

**CPET variables**	**Modified CD grade**		*P*
Grade <3 (Mild-Moderate) Mean ± SD	Grade ≥3 (Severe) Mean ± SD
VO_2_ peak			
Pre-chemotherapy	1115.2 ± 202.8	1187.5 ± 209.9	.498
Post-chemotherapy	1010.0 ± 199.3	1011.5 ± 195.0	.962
VO_2_ peak (mL min−1)			
Pre-chemotherapy	19.4 ± 2.9	19.5 ± 3.8	.734
Post-chemotherapy	17.4 ± 2.5	16.4 ± 2.4	.691
AT (mL min−1)			
Pre-chemotherapy	905.8 ± 184.3	911.5 ± 152.7	.161
Post-chemotherapy	780.6 ± 160.5	693.3 ± 140.9	.543
AT (mL kg min−1)			
Pre-chemotherapy	15.6 ± 2.2	14.8 ± 2.2	.981
Post-chemotherapy	13.4 ± 2.2	11.2 ± 1.1	.006
VE/VCO_2_	35.4 ± 5.1	33.1 ± 3.4	.387
Delta AT	2.2 ± 2.0	3.6 ± 2.4	.101

Values are given as mean ± SD. Table depicts change in exercise testing variables in comparison with severity of postoperative complications.

CPET, cardiopulmonary exercise testing; CD, Clavien–Dindo; VO_2_ peak, peak oxygen uptake; AT, anaerobic threshold; delta AT, change in anaerobic threshold.

**Table 5.** Univariate Analysis of Preoperative Variables and Complications

	**Modified CD classification**		*P*
Grade <3 (Mild-Moderate)	Grade ≥3 (Severe)
Age <60 ≥60	13 (48.1%) 14 (51.9%)	4 (66.7%) 2 (33.3%)	.656
Gender Female Male	10 (37%) 17 (63%)	3 (50%) 3 (50%)	.659
ASA 1 2 3	9 (33.3%) 15 (55.6%) 3 (11.1%)	1 (16.7%) 4 (66.7%) 1 (16.7%)	.712
Diagnosis Mid Lower	10 (37%) 17 (63%)	4 (66.7%) 2 (33.3%)	.363
Histology SCC AC	20 (74.1%) 7 (25.9%)	5 (83.3%) 1 (16.7%)	1.000
Chemotherapy TP 5FU-Cisplatin FLOT	19 (70%) 6 (22.2%) 2 (7.4%)	3 (50%) 3 (50%) 0 (0%)	.343
Surgical procedure Mckeowns Ivor Lewis	13 (48.1%) 14 (51.9%)	3 (50%) 3 (50%)	1.000
BMI Pre-chemotherapy Post-chemotherapy	21.7 ± 4.122.0 ±3.8	23.3 ± 6.123.5± 5.6	.607 .657

Values are given as percentages. Table depicts number and percentages of various variables in comparison with severity of complications.

CD, Clavien–Dindo; ASA, American Society of Anaesthesiologists; SCC, squamous cell carcinoma; AC, adenocarcinoma; 5FU, Fluorouracil; FLOT, 5-Fluorouracil–Leucovorin–oxaliplatin–docetaxel; AT, anaerobic threshold; TP, Carboplatin–Paclitaxel.
